# The Role of IgA in the Manifestation and Prevention of Allergic Immune Responses

**DOI:** 10.1007/s11882-023-01105-x

**Published:** 2023-08-23

**Authors:** Stephan Scheurer, Ann-Christine Junker, Chaoqi He, Stefan Schülke, Masako Toda

**Affiliations:** 1 Federal Institute for Vaccines and Biomedicines, Molecular Allergology, Paul-Ehrlich-Institut, Paul-Ehrlich Str., 51-58, 63225 Langen, Germany; 2 Laboratory of Food and Biomolecular Science, Graduate School of Agricultural Science, Tohoku University, Sendai, Japan; 3 Division of Allergology, Paul-Ehrlich-Institut, Federal Institute for Vaccines and Biomedicines, Langen, Germany

**Keywords:** Immunoglobulin A, IgA, Secretory IgA, Fc_a_RI, IgE-mediated allergy, Allergen-specific immunotherapy

## Abstract

**Purpose of Review:**

Immunoglobulin A (IgA) mediates immune exclusion of antigens in the gut. Notably, IgA plays also a role in the prevention of IgE-mediated allergies and induction of immune tolerance. The present review addresses the role of IgA in the manifestation of IgE-mediated allergies, including allergen-specific immunotherapy (AIT), the regulation of IgA production, and the mechanism of IgA in immune cell activation.

**Recent Findings:**

The majority of studies report an association of IgA with the induction of immune tolerance in IgE-mediated allergies. However, reports on the involvement of humoral and mucosal IgA, IgA subtypes, monomeric and polymeric IgA, and the mechanism of IgA-mediated immune cell activation are confounding.

**Summary:**

Effects by IgA are likely mediated by alteration of microbiota, IgE-blocking capacity, or activation of inhibitory signaling pathways. However, the precise mechanism of IgA-regulation, the contribution of serum and/or mucosal IgA, and IgA1/2 subtypes, on the manifestation of IgE-mediated allergies, and the underlying immune modulatory mechanism are still elusive.

## Introduction

IgE is well-known to play an essential role in the pathology of type I allergies. The induction of immune tolerance is mediated by the induction of IL-10-secreting Tregs and Bregs and likely associated with both the induction of allergen-specific IgG and of local and systemic IgA responses [1]. While the role of IgG in preventing allergic immune responses through its ability to compete with IgE epitopes as a blocking antibody and trigger inhibitory cell signaling pathways is well described, the role of IgA is less investigated. IgA is the most abundant antibody in the human body and is mainly secreted at mucosal surfaces. Two types of IgA exist, monomeric serum IgA and polymeric secretory IgA (SIgA) at the mucosal surface [2, 3].

The present review refers selected reports describing the engagement of serum and secretory IgA in the manifestation and prevention of IgE-mediated allergies and discusses potential mechanisms underlying the induction of IgA and its preventive effects.

## Structure of IgA

**Fig. 1:**
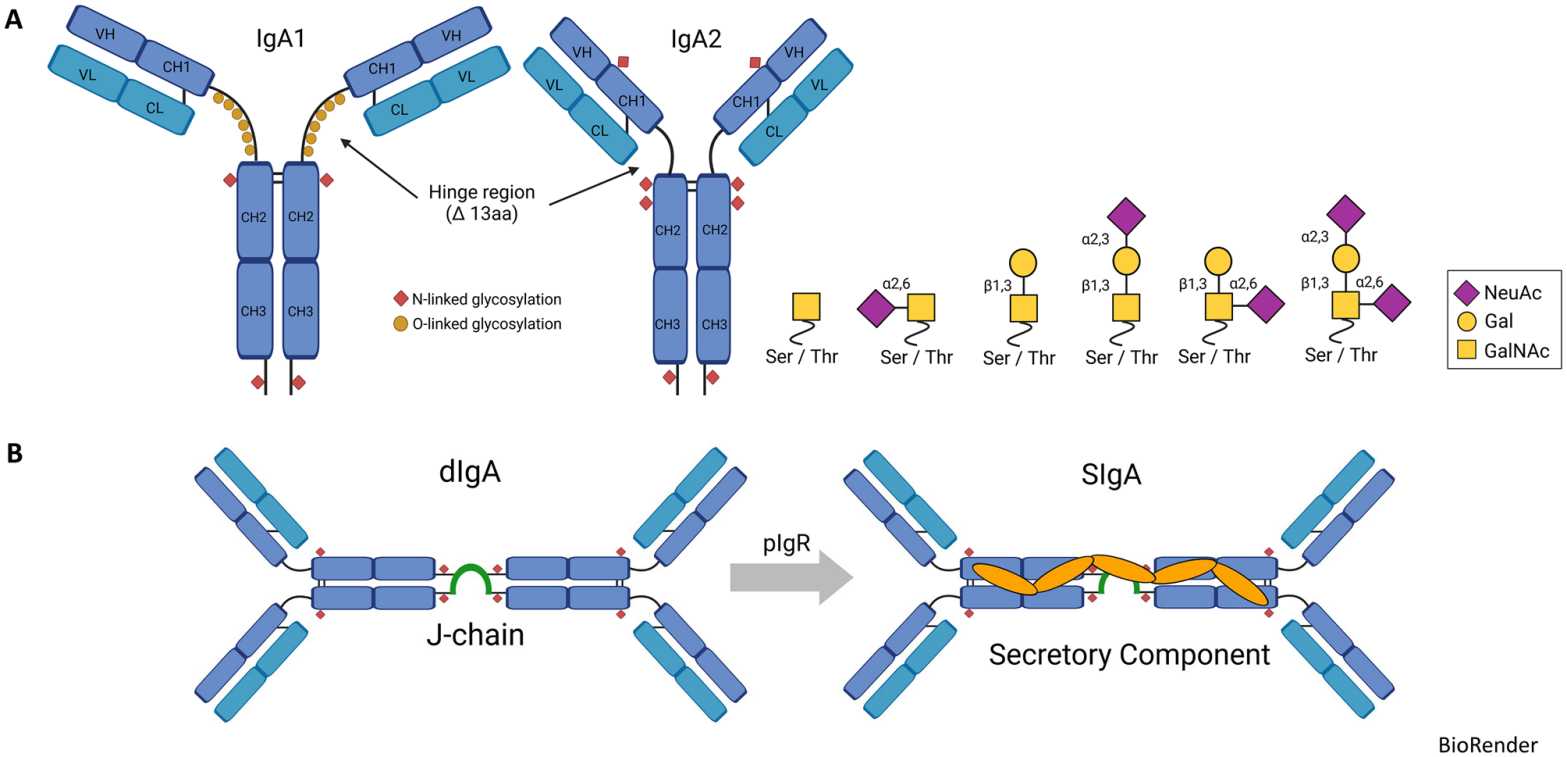
Schematic structure of IgA1, IgA2, and SIgA. **A** IgA1 expresses multiple O-glycosylation sites of serine and threonine in the hinge region, modified by either sialic acid (NeuAc), galactose (Gal), or N-acetylgalactosamine (GalNAc). IgA2 lacks the corresponding O-glycosylation sites in the hinge region and has only N-glycosylation sites modified by glucosamine (GlcN) and a lower level of sialylation. **B** Secretory IgA (SIgA) is generated from dimeric IgA (dIgA) upon dimerization via the 15-kDa J-chain in tissue plasma cells and binding to the pIgR on epithelial cells. Upon translocalization to the luminal site, the ectodomain of pIgR (secretory component, SC) is cleaved and stabilizes dIgA to form SIgA

In humans, two subtypes of IgA exist, IgA1 and IgA2, with predominance of the IgA1 subtype, e.g., 89% in serum and 70% in jejunal fluid [3]. IgA1 differs from IgA2 by additional 13 amino acids in the hinge region consisting of two repeated amino acid sequences [3]. These sequences possess multiple O-glycosylation sites of serine and threonine, which are highly modified by sialic acid, in addition to glycosylation by N-acetylgalactosamine (GalN) [4]. In contrast, IgA2 lacks O-glycosylation sites in the hinge region and reveals only N-glycosylation sites modified by glucosamine (GlcN) [4] and displays a lower level of sialylation [5]. Remarkably, glycosylation of IgA1 is highly heterogeneous [2], a property which has been considered to facilitate antigen recognition and to have a significant effect on its immune effector function [6]. The extended hinge region might also explain the increased susceptibility of IgA1 to bacterial proteases [2]. Interestingly, mice, rats, and most other mammals only express a single subclass of IgA that resembles human IgA2 [6] (Fig. 1A).

Secretory IgA (SIgA) is produced by plasma cells in the mucosa, including the gastrointestinal lymphatic tissue (GALT), and is important to sustain intestinal homeostasis by neutralizing pathogens due to immune exclusion [7]. SIgA is secreted as polymeric, predominantly dimeric IgA (dIgA) [2, 6]. Dimerization of IgA by a tail-to-tail conjugation is promoted by a conserved additional sequence termed joining chain (J-chain), which in turn consists of 18 amino acids at the C-terminal of the CH3 region at the IgA heavy chain [8], and which is important for transcytosis [9]. dIgA binds via the J-chain to the polymeric immunoglobulin receptor (pIgR) in the basolateral surface of epithelial cells and is internalized into endosomes. Next, it is transported via vesicles to the apical surface. At the luminal side, dIgA is proteolytically cleaved from the pIgR, and the ectodomain of pIgR called secretory component (SC) remains covalently attached to dIgA to form SIgA [10]. The SC fragment contributes to the stabilization of SIgA and allows further modification by glycosylation [2].

The highest levels of SIgA antibodies are generated and secreted at mucosal surfaces of the gastrointestinal, urogenital, and respiratory tracts, whereas systemic IgA antibodies in the bloodstream are found in lower concentrations. Approximately 3 g SIgA per day are produced in adult humans [11]. SIgA and IgM coat similar members of the microbiota present in the lumen. However, IgM occurs at a concentration nearly 100 times lower than that of IgA [11] (Fig. 1B).

## Regulation of IgA Production

Gut-associated lymphoid tissue (GALT) is the major site for IgA production. Peyer’s patches (PPs) are the most important inducing sites for T cell-dependent IgA1 class switching, whereas mesenteric lymph nodes (MLN) play a minor role in the IgA class switching during homeostasis [12]. The main site of T cell-independent IgA2 class switching is suggested to be the non-organized lymphoid tissue of the lamina propria in the colon [13, 14]. CCR9 and α4β7 integrin induce migration of IgA^+^ plasma cells from PPs to the lamina propria expressing CCL25 in small intestines [15]. During lactation, IgA^+^ plasma cells expressing CCR10 migrate from PPs into the mammary glands expressing CCL28 [16]. An axis of CCR10 and CCL28 is also involved in migration of IgA^+^ plasma cells to the bone marrow, where the cells are thought to release monomeric IgA antibodies into the bloodstream [17]. We briefly discuss the major molecules involved in the regulation of IgA production in the GALT and their link to allergy in this section.

### TGF-β

Under physiological conditions, transforming growth factor beta (TGF-β) plays a crucial role in both T cell-dependent and T cell-independent IgA production. TGF-β initiates the class switching process of IgA1 and IgA2 by inducing germline α-transcript expression in human B cells [12, 18]. For optimal IgA class switching, TGF-β signaling requires cooperation with other cytokines or molecules, such as IL-4, IL-5, IL-6, IL-10, IL-21, nitric oxygen (NO), and/or retinoic acid (RA). Successful AIT is attributed to increased levels of TGF-ß and Tregs, which potentially influence IgA levels in patients with allergies.

### Retinoic Acid

RA, a vitamin A derivative, is considered a secondary class switch recombination (CSR) factor that facilitates IgA isotype switching by creating a binding site for activation-induced cytidine deaminase (AID), in human B cells [19, 20]. The combined action of TGF-β and RA promotes IgA class switching and differentiation of human B cells into IgA-producing plasma cells [21]. Additionally, RA enhances the expression of α4β7 integrin and CCR9 on IgA^+^ plasma cells, promoting their migration from PPs to the lamina propria [22]. The primary source of RA in the GALT is CD103^+^ CD11c cells, a specialized subset of DCs [23]. CD103^+^ DCs take up dietary vitamin A metabolites, such as retinol, from the gut lumen and convert them into active RA by aldehyde dehydrogenases (ALDHs) [24]. CD103^+^ DCs also produce TGF-β that contributes to the induction of Treg cells and IgA production in B cells. Induction of CD103^+^ DCs appears to be a strategy to modulate allergy and IgA production.

### IL-21

IL-21 initiates the class switching of IgA1 and IgA2 by inducing germline α-transcript expression in human B cells [25, 26]. As for TGF-β, IL-21 preferentially induces the class switch to IgA1 (rather than to IgA2). The major source of IL-21 in the secondary lymphoid tissues is T-follicular helper (TfH) cells. A recent study suggested that IL-21 inhibits IgE production in both human and mouse B cells [26]. Accordingly, IL-21 could be a target molecule to reduce IgE and increase IgA in allergy treatment.

### BAFF and APRIL

B cell-activating factor (BAFF) and a proliferation-inducing ligand (APRIL) are members of the tumor necrosis factor (TNF) superfamily and induce class switching of human B cells to IgA1 and IgA2 [13]. Both molecules also play important roles in supporting B cell activation, survival, germinal center formation, CSR, and plasma cell differentiation [27]. A recent study showed that APRIL promotes the differentiation of naïve human B cells into IL-10-producing IgA^+^ B cells upon continuous exposure to CD40L and IL-21 [28]. In GALT, intestinal EC, macrophages, and stroma cells are the major sources of APRIL and BAFF. It remains to be elucidated whether and how the expression of BAFF and APRIL in the GALT interacts with allergy and IgA.

### Microbiome and its Metabolites

The critical role of the gut microbiome and their signals via TLRs has been well established in the regulation of IgA production in the intestinal mucosa. Germ-free (GF) conditions significantly reduce intestinal IgA-secreting cells and IgA production, whereas transfer of commensal bacteria restores IgA production [29]. The engagement of TLRs and BCRs by microbiome-derived molecules is an essential step in T cell-independent IgA production in B cells. TLR signaling in DC, macrophages, IECs, and other cells induces the expression of cytokines, including TGF-β, APRIL, and BAFF.

Short-chain fatty acids (SCFAs) are the major anti-inflammatory bacteria-derived metabolites in the gut and also involved in IgA production. Among SCFAs, acetate promotes vitamin A metabolism in IECs and DCs via GPR43 that in turn enhances production of luminal IgA [30] SCFAs can also directly promote B cell differentiation into IgG- or IgA-secreting plasma cells [31]. A recent study showed that acetate orchestrated the interactions between epithelial and immune cells and induced microbially stimulated CD4^+^ T cells to support T cell-dependent IgA production for certain types of microbiomes and, as a consequence, altered the localization of these bacteria within the colon [32]. Evidence has accumulated that dysbiosis and reduced intestinal levels of SCFA are associated with the development of food allergies [33]. Since lower levels of IgA in serum have also been observed in patients with food allergies (third section), it will be interesting to investigate whether stool IgA levels are linked to dysbiosis and to disease status.

Recent advances in IgAseq have enabled the identification of IgA-coated gut bacteria. IgA broadly recognizes the microbiome via low affinity by binding to various glycans, LPS, and flagellin. Low-affinity IgA contributes to the maintenance of commercial bacteria, whereas high-affinity IgA binds to pathogens to inhibit the interaction with host and promote clearance [14, 34]. The disease state can also alter the binding profile of IgAs against gut bacteria. Palm et al. showed that a subset of highly IgA-coated intestinal microbiota from patients with inflammatory bowel disease selectively confers susceptibility to colitis [35]. In patients with multiple sclerosis, immunostimulatory gut bacteria lead to an expansion of regulatory IgA-secreting plasma cells that were detected in their inflamed central nervous system [36]. It remains to be elucidated whether the presence of highly IgA-coated bacteria could be a signature in either development, exacerbation, or remission of allergy.

Interestingly, an association between gut microbiota, IgA, and allergy is described. Allergic children not only had low salivary SIgA levels but also a less differentiated bacterial microenvironment [37]. Patients with IgA deficiency (SIgAD) have an altered gut microbiota composition compared to healthy patients [37]. Although direct effects of gut SIgA on the allergic response remain elusive, it is tempting to speculate that modulation of SIgA production by targeting the microbiota, e.g., by prebiotics, may modulate allergy. Evidence has accumulated that dietary fibers, e.g., pectin, promote IgA production (own unpublished data). However, the reports on the effect of pectin diet on SIgA levels are controversial. [38–40]. Remarkably, pectin has been suggested to change the Th1/Th2 balance towards Th1 immunity [41].

## Role of IgA in Allergic Diseases

The importance of IgA for the prevention of allergies can be deduced from breastfeeding, patients with IgA immunodeficiency, and therapeutic intervention studies.

### IgA Response and Manifestation of Allergies

Maternal IgA is primarily secreted across mucosal barriers or into breast milk, providing an immune barrier at these interfaces and contributing to protection against infections. Moreover, IgA in breast milk plays an important role in the prevention of cow’s milk allergy (CMA) in the offspring [42, 43]. Strong evidence was provided that total IgA, rather than cow’s milk (CM) allergen-specific IgA, in breast-fed infants promotes protection against the development of CMA [44]. Moreover, maternal avoidance of CM was associated with lower CM-specific IgA in both mothers and infants and with higher prevalence of CMA in infants [45]. The PASTURE study showed the levels of total IgA in breast milk and the amount of IgA ingested to be inversely associated with the risk of developing atopic dermatitis (AD) in infants [46].

The relevance of IgA is further reflected by the natural course of tolerance induction [47]. High concentrations of IgA in stool and serum have been associated with protection against IgE-mediated allergic diseases. In line with this, high intestinal IgA in early infancy at the age of six months was associated with a reduced risk for IgE-associated allergic diseases before the age of 2 years [48]. In line with this, serum egg white-specific IgA2 levels were shown to be approximately 4 times lower in egg-allergic children than in non-allergic controls and increased by 28% in those who developed natural tolerance [49].

However, a recent study reported that mucosal IgA present in the gut, in contrast to systemic IgA responses, does not provide protection against food allergies [50]. The authors found that allergen-specific IgA did not correlate with natural tolerance to food allergens. In this study, 512 IgE-sensitized but peanut-tolerant patients were monitored over 2 years. No significant association of peanut-specific gastrointestinal IgA values with peanut-specific IgE and manifestation of clinical allergy was observed. Surprisingly, serum IgA values were not associated with peanut-specific IgE and the development of allergy. The current study reveals that peanut (and egg white-)-specific, gut IgA may not be a marker of food tolerance and that IgA specificity cannot be used to discriminate between allergic and tolerant individuals. The authors showed that peanut-specific gut IgA recognizes different epitopes than peanut-specific serum IgE. Importantly, these findings indicate that epitope specificity of peanut-specific gut IgA does not distinguish between children with and without a peanut allergy. These assumingly conflicting results about the role of IgA were recently discussed by Seppo et al. [47]. The study by Liu et al. [50] refers to a retrospective study (CoFAR2), comprising of both sensitized and/or allergic children with a broad age range (3–15 months), but allergic sensitization occurs often by 4–6 months of age, which might raise confounding effects. Seppo et al. [47] also suggested that in addition to the time course of IgA production, at which atopy may be associated with a transient delay in IgA immunity, the location of induced IgA response is important. It remains elusive whether the IgA level and the ratio of IgA isotypes in fecal samples reflect the quantity of IgA and IgA isotypes seen in the small intestine at the site of the local immune response. Studies to elucidate the mechanisms of IgA and mucosal IgA^+^ plasma cells at the actual site of action, such as the small intestinal gut mucosa, are required but are difficult to perform.

### Selective IgA Deficiency (SIgAD)

SIgAD is a primary immunodeficiency characterized by low levels of serum IgA (< 0.07 g/L), while other antibody isotypes are expressed at normal values. The association of SIgAD with the manifestation of allergies is controversially discussed [37]. However, most of the studies reported an association between SIgAD and the manifestation of allergies, atopic rhinitis, atopic dermatitis or asthma, and food allergy [51]. SIgAD develops in the first 4 years of age and, although asymptomatic in the majority of affected individuals, frequently is accompanied by clinical symptoms due to infections and allergic diseases [37, 52]. Besides decreased levels of serum IgA, SIgAD patients express even low levels of secretory IgA which was suggested to facilitate allergen transfer across the mucosa and increase allergic sensitization in approximately 40% of SIgAD patients [37]. This suggests that decreased serum IgA antibody levels might predispose to an increased intestinal mucosal permeability and absorption of ingested antigens, therefore increasing the risk of severe food allergy [37]. However, it remains unclear whether SIgAD promotes allergy or is a secondary effect resulting from a diminished mucosal activity due to the allergic reactions [37].

### IgA Responses Affected by AIT

The effect of AIT on the IgA response has been frequently reported [53, 54]. Early studies by Platts-Mills et al. [55] provided evidence that ragweed allergen antigen E (AgE)-specific nasal IgA increased substantially after AIT, supporting the hypothesis that IgA has certain allergy protective capacity. However, contradictive data were obtained as patients with ragweed allergy had higher IgA values per se than ragweed tolerant patients.

Jutel et al. [56] reported a significant increase of Der p 1-specific serum IgA (and IgG4) after HDM-SCIT. Increased IgA production was attributed to increased Treg-derived TGF-ß which acts as Ig isotype switch factor and likely contributed to clinical efficacy [56]. Remarkably, baseline-specific IgA values were high in both the healthy control group and also in allergic patients. However, the study did not dissect IgG4- and IgA-mediated effects. In an independent study, the antibody response during SLIT in HDM allergic pediatric patients was monitored [57]. In contrast to the previous study, Der p 1-specific baseline IgA levels in *serum* of the allergic group were significantly lower than that of the healthy controls. Specific IgA increased over 12 months SLIT but without reaching significance compared to baseline, with similar values observed in the healthy control group at the end of the SLIT [57]. However, mucosal IgA response and IgA subtypes were not determined in the study. The authors suggested to further investigate the role of TGF-ß and Tregs during SLIT.

So far, the IgA response to AIT has been less characterized with regard to its subclass distribution, production site, and its relationship to the expression of TGF-ß. Pilette et al. [58] showed grass allergen-specific IgA2, but not IgA1, in serum to be selectively induced after 2 years of double-blind grass pollen SCIT and was accompanied by nasal TGF-ß mRNA expression. Notably, the IgA2-containing serum fraction did not compete the IgE-facilitated grass allergen presentation by B cells. Serum fractions containing polymeric IgA2 (pIgA2) were used to passively sensitize autologous monocytes. Subsequent in vitro cross-linking of pIgA2 on monocytes by antigen or anti-IgA resulted in IL-10 production, supporting a further role for IgA for induction of tolerance. A later study showed both grass pollen-specific IgA1 and IgA2 antibody levels to be slightly but significantly elevated during the peak pollen season in the SLIT-treated group when compared with baseline [59]. The importance of IgA was further supported in patients with clinical response to egg OIT showing increased EW-specific IgA:IgE and IgA2:IgE ratios in serum, accompanied by a relative increase of specific IgG4 in responders versus non-responders [60]. Importantly, these studies dissect between IgA1 and IgA2 responses. The authors concluded that serum IgA2 rather than IgA1 is associated with positive AIT. It has been suggested that IgA2 likely reflects IgA- and TGF-ß production at mucosal surfaces, and SIgA in the gut lumen likely will not have a protective effect.

In contrast to previous studies, the local IgA response was monitored in a peanut SLIT study [61]. Importantly, peanut-specific secretory IgA in the saliva increased in subjects who responded favorably to peanut SLIT. Later, Smeekens et al. [62] studied saliva mucosal IgA in response to peanut OIT. Both peanut-specific IgG4 and peanut-specific IgA/total IgA ratios significantly increased during OIT. Without experimental proof, salivary IgA was considered to reflect the amount of peanut-specific IgA present at the gastrointestinal mucosal surface. The authors speculated high levels of mucosal IgA to intercept the peanut antigen, preventing uptake by tolerogenic dendritic cells and impede tolerance induction. However, the hypothesis was not supported from mouse models of egg allergy, where serum IgA, but not secretory gastrointestinal IgA, was essential in protecting mice from anaphylaxis after the oral administration of egg [63].

Recently, a head-to-head (GRASS) trial comparing for the first time the humoral immune response after SLIT and SCIT was performed. Results showed that SCIT induces largely allergen-specific serum IgG4 but only moderate systemic IgA1 and IgA2 responses, whereas SLIT significantly induces both IgA1 and IgA2 in nasal fluid and IgA1 in serum [64••]. These antibodies increased after 2–6 months during AIT and were detectable both in serum and in nasal fluid, with IgA2 values approximately threefold over baseline after 2 years of SLIT [64••, 65]. Remarkably, elevated IgA levels persisted 1 year after discontinuation of SLIT and allergic symptoms correlated inversely with IgA1 in nasal fluid. Shamji et al. pointed out that naturally occurring allergen-specific IgG and IgA recognize epitopes distinct from IgE, whereas upon AIT, both Ig subclasses can compete with IgE binding [65]*.*

## Molecular Mechanism by Which IgA Affects Allergic Immune Responses

SIgA has important effector and regulatory functions in order to maintain a delicate balance in mucosal immunity: (1) immune exclusion at the luminal side [3, 66–68], blocking colonization, and penetration of pathogenic microorganisms without causing chronic inflammation at mucosal barriers and (2) neutralization of pathogens via induction of antigen-specific effector responses including phagocytosis, antibody-dependent cellular cytotoxicity, superoxide generation, release of inflammatory mediators, and cytokines as well as antigen presentation [69–71].

### Anti-inflammatory Functions Involved in the Maintenance of Tolerance and Suppression of Allergic Responses

Immunological changes associated with successful AIT include the induction of allergen-specific antibodies, mainly IgG but also IgA that are supposed to (1) block the interaction of allergen with IgE, (2) prevent the activation of mast cells and basophils, and (3) reduce the IgE-dependent uptake, maturation, and presentation of allergen by antigen-presenting cells (APCs) [1, 37, 53].

In agreement for IgG, an association of IgA with the induction of IL-10 has been reported: monocytes produce the anti-inflammatory cytokine IL-10 after IgA-mediated activation, which inhibits IL-6 and TNF-α production [72]. Noteworthy, polymeric IgA2 purified from post-immunotherapy serum used to passively sensitize autologous monocytes triggered IL-10 and TGF-β production upon allergen-mediated cross-linking in vitro [58]. In line with this, a subset of blood-derived IgA^+^ regulatory B cells producing IL-10 and expressing PD-L1 and Fas-L was described both in humans and mouse models of experimental autoimmune encephalitis and contact hypersensitivity [28].

The preventive effect of IgA on allergic sensitization was suggested to be mediated (1) by exclusion of allergen transfer across offspring gut [1], (2) by affecting effector cells, and (3) the intestinal flora [73]. However, the exact contribution of allergen-specific IgA to either the development or prevention of allergies remains unknown.

Food-specific IgA antibodies were hypothesized to bind to the respective antigen in the gut lumen, thereby preventing antigen absorption via immune exclusion while promoting their digestion by intraluminal proteolytic enzymes [74].

In line with the theory that IgA may interfere with mast cell activation, Strait et al. reported both serum antigen-specific IgG and IgA antibodies to suppress IgE-mediated food allergy in mouse models of active and passive sensitization with systemic IgA providing better protection than enteric IgA [63]. In a very recent study, El Ansari et al. [75••] reported IgA to bind to both mouse bone marrow-derived mast cells and peritoneal mast cells in a calcium- and sialic acid-dependent manner. IgA was found to inhibit allergen-specific, IgE-dependent mast cell degranulation by suppressing both phosphorylation of Syk and mast cell cytokine production [75••]. IgA was also able to suppress activation of human basophils isolated from a peanut allergic donor [75••]. These results further support a role of IgA in the regulation of mucosal homeostasis by controlling the receptor-mediated activation of mast cells and basophils. While the authors suggested the suppressive effects of IgA to be mediated by a specific (likely C-type lectin) receptor rather than a steric blockage of the IgE epitopes on the allergen molecules, the identification of the responsible receptor is elusive [75••].

### IgA-Binding Receptors

In order to understand why polymeric SIgA and monomeric serum IgA induce different responses, IgA-binding receptors and IgA-mediated signaling events need to be explored in more detail. IgA can bind to various receptors, preferentially to pIgR and FcαRI (CD89), but also to FcRL4 (CD307d), Fcα/μ receptor (CD351), transferrin receptor (CD71), and lectin receptors like the asialoglycoprotein receptor (ASGPR), dectin-1 (CD369), and DCSIGN (CD209) [2]. Here, modification of IgA by glycan residues in either the Fc domain, SC, or J-chain can facilitate receptor binding [76–78].

The contribution of these IgA receptors to the overall immune responses remains only partly understood. Interestingly, both the inductions of IgA-dependent pro- and anti-inflammatory signaling seem to be mainly mediated via FcαRI [79]. In humans, FcαRI (encoded by the FCAR gene) is constitutively expressed on myeloid cells, predominantly on the surface of neutrophils, but also on eosinophils, monocytes, and macrophages, and likely also on platelets, but not on either mast cells, basophils, or intestinal macrophages [80]. In allergic patients, an enhanced expression of FcαRI was described on eosinophils but not on neutrophils [81]. FcαRI displays a higher sequence similarity with KIR and LIR found on NK cells than with other Fc receptors [76–78]. Remarkably, mice lack FcαRI [2], whereas a CD89 homolog was described in rats [82]. In mice, CD351 (expressed on macrophages, follicular DC/B cells, kidney tubular epithelial cells) is the only receptor known to bind IgA.

### Contribution of IgA Subclasses to Pro- and Anti-inflammatory Immune Responses

Glycosylation of IgA is both highly heterogeneous and associated with many diseases, e.g., autoimmune diseases such as IgA nephropathy, IgA vasculitis, systemic lupus erythematosus, and rheumatoid arthritis [6]. Different glycosylation patterns between IgA1 and IgA2 modify the strength of IgA interactions with FcαR [83, 84]. However, the involvement of IgA glycosylation in the development of allergic Th2 responses is still unclear.

Steffen et al. [85••] reported the effector function of IgA to depend on both IgA subclass and glycosylation: while IgA2 with lower levels of sialylation effectively promoted pro-inflammatory responses (NET formation and cytokine secretion from neutrophils), IgA1 with higher levels of sialylation did not have comparable effects [85••]. In line with these results, enzymatic removal of either the sialylation or the complete N-glycans significantly increased the pro-inflammatory capacity of IgA1 to levels that were observed for IgA2 [85••]. Concordantly, in rheumatoid arthritis patients, disease-specific autoantibodies were shown to shift towards the pro-inflammatory IgA2 subclass, correlating with disease progression [85••].

### Pro- and Anti-inflammatory Signaling Mediated by the FcαRI

Binding of antigen to specific IgA can result in the formation of immune complexes which bind to immune cells via FcαRI. Fascinatingly, binding of either monomeric IgA or polymeric SIgA to FcαRI triggers distinct signaling events. However, a single FcαRI alone cannot trigger intracellular signaling, as its short cytoplasmic tail lacks the necessary signaling motifs [69] and therefore needs to associate with other receptors (that provide a second signal) in order to either amplify or inhibit the immune cell activation [86]. This inability of single IgA-binding to FcαRI to trigger inflammatory responses by itself is considered an important factor in limiting potentially detrimental inflammatory responses induced by IgA.

### Signaling Events Leading to Pro-inflammatory IgA-Mediated Signaling

**Fig. 2:**
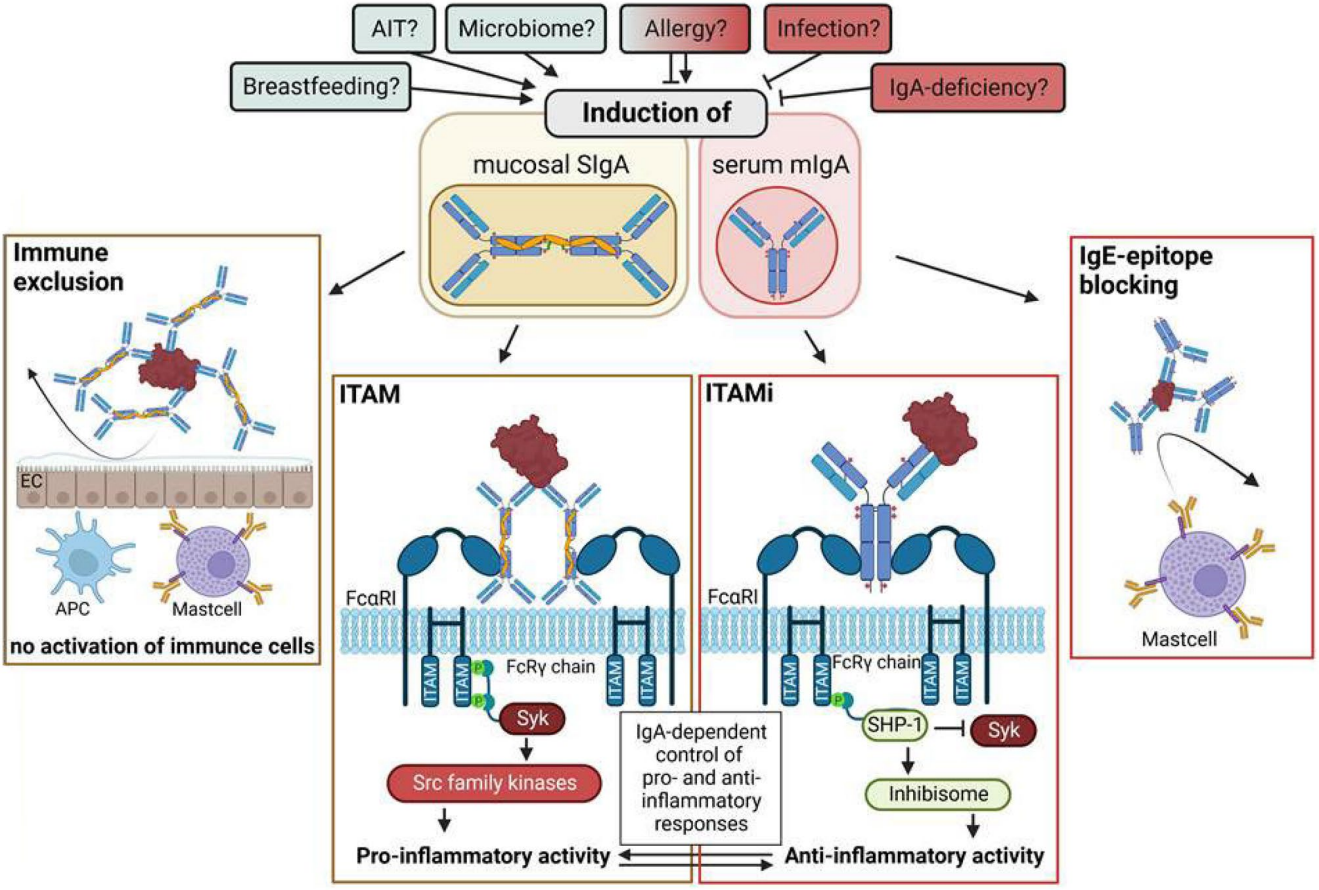
Modulation of (allergic) immune responses by IgA. IgA production can be modulated by different factors such as the microbiome, certain infections, breastfeeding, genetic defects in IgA production, allergic sensitization, or AIT. The exact contribution of these factors to either the production of SIgA in secretions and monomeric IgA in serum remains unclear. Mucosal SIgA can prevent allergen absorption and promote allergen digestion via immune exclusion which prevents inflammatory responses towards the respective allergens. Moreover, mucosal SIgA can form aggregates with allergens in secretions that trigger pro-inflammatory signaling and effector functions via a FcαRI-dependent recruitment and activation of Src family kinases. In contrast, monomeric IgA in serum only results in an initial low-intensity activation of FcαRI by partial phosphorylation of FcRγ ITAM tyrosines. This promotes a transient recruitment of Syk followed by stable recruitment of SHP-1 to the FcRγ ITAM, the formation of intracellular “inhibisomes,” and the induction of anti-inflammatory responses. In addition, IgA can compete with IgE for allergen binding and thereby diminish IgE-mediated effector cell activation

Sustained aggregation of FcαRI by immune complexes containing SIgA results in immune cell activation, characterized by phagocytosis, antigen presentation, production of reactive oxygen species, Ab-dependent cellular cytotoxicity, and cytokine release. In order to initiate pro-inflammatory signaling events, FcαRI must associate with immunoreceptor tyrosine-based activation motif (ITAM) motifs. Binding of antigen:IgA immune complexes to FcαRI triggers the intracellular recruitment of Src family kinases and phosphorylation of tyrosine residues in the ITAM motif-containing FcRγ [87–90] (Fig. 2).

### Signaling Events Leading to Anti-inflammatory IgA-Mediated Signaling

In contrast to the above described functions of SIgA in mucosal secretions, monomeric IgA in serum displays immunological functions different from secretory IgA such as anti-inflammatory activity, inhibiting IgG-induced phagocytosis, bactericidal activity, oxidative burst, or cytokine release [3] (Fig. 2).

The affinity of monomeric IgA (without antigen) to FcαRI is low. However, binding of monomeric, uncomplexed IgA to FcαRI does not lead to cross-linking but instead triggers anti-inflammatory signaling events. This inhibitory signaling triggered by IgA involves recruitment of the tyrosine phosphatase SHP-1 to FcαRI and impairment of Syk, LAT, and ERK phosphorylation [87]. In this context, initial low-intensity activation of FcαRI by partial phosphorylation of FcRγ ITAM tyrosines promotes a transient recruitment of Syk followed by stable recruitment of SHP-1 to the FcRγ ITAM [91]. FcαRI associated with FcRγ cluster in lipid rafts, triggering endocytosis and the formation of intracellular “inhibisomes” in which SHP-1 dephosphorylates signaling molecules associated with cell activation [91] (Fig. 2).

Alternatively, binding of IgA via glucan residues to dendritic cell-specific intercellular adhesion molecule-3-grabbing non-integrin (DCSIGN) and specific ICAM-3 grabbing nonintegrin-related 1 (SIGNR1) can control inflammatory responses via a DC-dependent induction of regulatory T cells [92].

By this complex dichotomy in IgA-mediated signaling events, IgA can have both pro- and anti-inflammatory functions contributing to the exclusion and elimination of pathogens while at the same time maintaining tolerance towards harmless microorganisms and environmental stimuli (Fig. 2).

## Summary

The distinctive feature of IgA in humans is that it exists in structurally different forms, either as a monomer or as a polymer, depending on whether IgA is induced systemically or locally at the mucosal tissue. In addition, there are two subclasses IgA1 and IgA2, which differ mainly by strong sialylation of IgA1. It should be noted that due to the absence of these two differently glycosylated IgA subclasses and the lack of expression of IgαRI in mice, the transfer of results obtained in mice to humans must be critically discussed.

Most studies on maternal IgA transfer and IgA deficiency demonstrate the inverse relationship between serum and secretory IgA levels and the development of allergy. IgA induction over time of natural tolerance induction is controversial in this regard and needs further investigation. Moreover, several clinical studies confirmed the induction of specific serum IgA during AIT and the induction of tolerance. However, only some studies dissected IgA1 and IgA2 responses, but with controversial results regarding the significance of the subclasses.

Of particular interest is the study by Shamji et al. [64••], who evaluated SLIT and SCIT side by side with respect to IgA1 and IgA2 levels in both serum and nasal fluid. They concluded that IgA1 was associated with a reduction in clinical symptoms with SLIT. Although there is some evidence that SCIT is preferentially associated with IgG4, whereas SLIT is associated with induction of IgA in both serum and nasal fluid, further conformational studies are needed to investigate possible differences in IgA induction and function depending on the type of AIT.

Accordingly, in subjects undergoing AIT, studies addressing the correlation of IgA with IgG4 induction and clinical symptoms are needed, as well as studies systematically monitoring IgA1 versus IgA2, specific versus total IgA, and systemic versus local IgA responses to further evaluate the role of IgA in the induction of tolerance.

Current data suggest that the preventive function of IgA is mediated by humoral allergen-specific IgA, although the roles of IgA subclasses and the contribution of mucosal IgA induction need to be evaluated. Future studies should consider that secretory IgA2 dimers are functionally more resistant to proteolytic cleavage by mucosal proteases than secretory IgA1.

Furthermore, the underlying molecular mechanisms of how IgA exerts its preventive effect remain unknown. For example, possible functional differences between systemic and local IgA should be explored. Several questions remain to be answered: does specific IgA in serum block IgE binding to high-affinity (FcεRI) and low-affinity (CD23) receptors for IgE expressed on antigen-presenting cells and basophils/mast cells, and does it inhibit IgE-mediated cross-linking and subsequent activation of effector cells? To what extent does the binding of IgA to inhibitory receptors mediate the immunomodulatory effect? In this regard, El Ansari et al. [75••] have recently shown that IgA-mediated inhibition of mast cell activation is receptor-mediated and depends on calcium and sialic acid modification of IgA. Therefore, it is reasonable to speculate that highly glycosylated IgA1 rather than IgA2 preferentially contributes to the observed effects. Since mast cells and basophils do not express IgAR (CD89), IgA is likely recognized by as yet unknown C-type lectin receptor. The authors [75••] however report that targeted deletions of C-type lectin receptors expressed on mast cells (including SIGN-R1, CD33, and Siglec F) and FcyRIIb did not affect IgA-mediated inhibition of IgE-dependent cell activation (El Ansari et al., data not shown).

However, the effect of allergen-specific IgA signaling on CD89 expressing cells is less investigated. Accordingly, it remains unclear by what mechanism ITAMi or ITIM mediate inhibitory signals. According to Steffen et al. [85••], the effector function of IgA depends on both IgA subclass and glycosylation: whereas IgA2 with lower levels of sialylation effectively promoted pro-inflammatory responses, and IgA1 with higher levels of sialylation had no comparable effects [85••]. It remains open whether this hypothesis is valid not only for monomeric but also for secreted IgA. When investigating this question, it should be taken into account that specific IgA bound on the receptor can be cross-linked by corresponding allergens with IgA bound to a second IgαR, but probably also with allergen-specific IgE bound to IgεR, or with allergen-specific IgG bound to FcγRIIb to further modulate immune responses.

Finally, to explore the engagement of IgA, its subclasses, structure, and modification by glycosylation in the manifestation of allergic immune response will be a challenging task in the future.

## Abbreviation

AID: Activation-induced cytidine deaminase
ALDHs: Aldehyde dehydrogenases
APCs: Antigen-presenting cells
AID: Activation-induced cytidine deaminase
APRIL: Proliferation-inducing ligand
BAFF: B cell-activating factor
CMA: Cow’s milk allergy
CSR: Class switch recombination
dIgA: Dimeric IgA
EC: Epithelial cells
EW: Egg white
GalN: N-Acetylgalactosamine
GALT: Gastrointestinal lymphatic tissue
ITAM: Immunoreceptor tyrosine-based activation motif
J-chain: Joining chain
MLN: Mesenteric lymph nodes
PPs: Peyer’s patches
pIgR: Polymeric immunoglobulin receptor
RA: Retinoic acid
SCFA: Short-chain fatty acids
SIgA: Secretory IgA
SC: Secretory component
SIgAD: Selective IgA deficiency
TGF-β: Transforming growth factor beta
TNF: Tumor necrosis factor
